# Tempering Expectations on the Medical Artificial Intelligence Revolution: The Medical Trainee Viewpoint

**DOI:** 10.2196/34304

**Published:** 2022-08-15

**Authors:** Zoe Hu, Ricky Hu, Olivia Yau, Minnie Teng, Patrick Wang, Grace Hu, Rohit Singla

**Affiliations:** 1 School of Medicine Queen's University Kingston, ON Canada; 2 School of Biomedical Engineering University of British Columbia Vancouver, BC Canada; 3 School of Medicine University of British Columbia Vancouver, BC Canada; 4 Temerty Faculty of Medicine University of Toronto Toronto, ON Canada

**Keywords:** medical education, artificial intelligence, health care trainees, AI, health care workers

## Abstract

The rapid development of artificial intelligence (AI) in medicine has resulted in an increased number of applications deployed in clinical trials. AI tools have been developed with goals of improving diagnostic accuracy, workflow efficiency through automation, and discovery of novel features in clinical data. There is subsequent concern on the role of AI in replacing existing tasks traditionally entrusted to physicians. This has implications for medical trainees who may make decisions based on the perception of how disruptive AI may be to their future career. This commentary discusses current barriers to AI adoption to moderate concerns of the role of AI in the clinical setting, particularly as a standalone tool that replaces physicians. Technical limitations of AI include generalizability of performance and deficits in existing infrastructure to accommodate data, both of which are less obvious in pilot studies, where high performance is achieved in a controlled data processing environment. Economic limitations include rigorous regulatory requirements to deploy medical devices safely, particularly if AI is to replace human decision-making. Ethical guidelines are also required in the event of dysfunction to identify responsibility of the developer of the tool, health care authority, and patient. The consequences are apparent when identifying the scope of existing AI tools, most of which aim to be physician assisting rather than a physician replacement. The combination of the limitations will delay the onset of ubiquitous AI tools that perform standalone clinical tasks. The role of the physician likely remains paramount to clinical decision-making in the near future.

## Introduction

The field of artificial intelligence (AI) in medicine has seen rapid development in the last decade, with an increasing number of applications introduced in clinical settings [1]. With the rapid growth in computing power and data, medical AI has transformed from an afterthought into an imminent possibility.

Currently, the utility of AI in completing tasks such as diagnostic prediction, automation, and generation of features from clinical data is recognized in many specialties. Models predicted the incidence of myocardial infarction and outperformed the current gold standard American College of Cardiology and American Heart Association risk algorithm [2]. These technological advancements have understandably raised concerns among health care trainees and professionals that AI may be taking over their duties. A study assessing medical students’ views regarding the impact of AI on future careers reported that 78.77% (1707/2167) expect significant changes due to AI and 89.62% (1942/2167) expressed that careful supervision by humans is required [3].

To moderate the concerns of AI in disrupting the future role of physicians, an understanding of the capabilities and limitations of AI tools is required. Wiens et al [4] reported AI adoption challenges, including problem formulation to market transition, all of which will require cooperation with interdisciplinary teams and systemwide change. In addition to refining the results of an AI algorithm, how the results are conveyed must also be accepted. Even if a physician accepts the judgement of a computer as legitimate, patients may not be nearly as receptive.

The aim of this commentary is to analyze the multifaceted issue of medical AI adoption to temper preconceived notions regarding its impact and rapid progression. We identify and explore four major barriers to AI adoption: (1) the limitations of performance and biases in AI applications, (2) the limitations due to heterogeneous digital infrastructure, (3) the limitations due to lack of technological literacy, and (4) the limitations of ethical challenges associated with medical AI usage.

## Limitations of Performance

A significant barrier for AI applications to be implemented is regulatory approval, such as by the Food and Drug Administration (FDA), where AI applications would be included in the recently created category of Software as a Medical Device [5]. Certification is required for a recognized regulatory body to approve of a device’s safety and effectiveness. If a new medical device is not considered a low- or moderate-risk device, it is required to enter the stringent premarket approval pathway, where demonstration of safety and effectiveness is required from clinical studies. The device is also classified in risk classes from Class I (the lowest risk) to Class III (the highest risk) [5]. AI, particularly machine learning, poses unique challenges as a machine learning model may continuously update with new training data. As such, the FDA has created recent guidelines, indicating that surveillance is required over the total product life cycle of the device, including model updates from retraining [6].

A standalone diagnostic tool would likely enter the premarket approval pathway and require extensive testing such as randomized controlled trials [7]. Leeuwen et al [8] evaluated 100 AI devices with CE-marked approval in Europe and reported that only 2 products were classified as class III, requiring premarket approval. Of 100 AI devices, 64 had no peer-reviewed studies validating the product performance. Wu et al [9] evaluated 54 AI medical devices approved by the FDA, with none being standalone diagnostic devices without physician supervision and none tested in a prospective trial. Hence, the current state of AI devices toward the FDA label of Computer-Assisted Detection Devices, which pose less resistance for market entry. The financial incentive results in a trend of devices being developed as physician-assisting tools that physicians can use at their discretion [10].

A technical barrier for AI devices to replace human analysis is the current performance of AI devices. For instance, when validated on a data set from a single center, convolutional neural networks (CNNs) routinely achieve accuracies above 0.90 [11]. However, with the variability of medical imaging from different machines, operators, or imaging protocols, multicenter studies are required to validate the generalizability of these classifiers. Alice et al [11] reported that 81% of diagnostic algorithms reviewed results in significant decrease of accuracy when externally validated. Thus, rigorous validation is required with a diverse data set to address the major machine learning challenges of data scarcity, population shifts from different data sets, prevalence shifts, and selection biases [12]. External validation also reveals a more accurate comparison between human and machine performance. Rodriguez-Ruiz et al [13] reported that when testing a published CNN to classify malignancies from mammography on a data set of 2652 images from seven different countries, the CNN performed within the same 95% CI accuracy range of 101 different radiologists [13].

The rigorous validation requirements for AI to be usable in clinical practice is evident when analyzing rapidly developed AI models. In the COVID-19 pandemic, over 100 diagnostic prediction models have been trained and published in literature, using features such as chest x-ray data, lung ultrasound, vital signs, and lab values. The reported concordance index of such models ranged from 0.71-0.99. However, Wynants et al [14] assessed that only 5% of the models found performed external validation, and only 2 models addressed selection biases during sampling.

An additional challenge for AI applications is that the ability to learn complex features is restricted to the architecture of the AI model. For instance, medical applications for CNNs commonly use architectures that perform well on the ImageNet challenge. The CNN architecture defines model parameters such as resolution, depth, and number of input channels, all of which affect the ability to detect complex features related to some objective. However, newer architectures are frequently developed, such as EfficientNet outperforming ResNet, DenseNet, Xception, and ResNeXT, all of which have been previously used in medical image classifiers [15]. Updating the model architecture is a significant change to the model. For instance, ResNet introduces the usage of residual blocks in a layer as an input for a subsequent layer to begin learning, changing how the model is initialized. This may require reapproval from regulatory bodies due to nontrivial changes in the device.

The alternative of a physician-assisting device is more likely in the near future, such as automating report extraction from imaging studies or image reconstruction to reduce excessive radiation from repeated imaging [16,17]. This reduces competition with physician tasks while still providing clinical utility from complex AI analyses.

## Limitations of Current Infrastructure

Implementation of an AI product, even with validated performance, is limited by heterogenous digital infrastructure in health care systems. Different areas of patient care such as inpatient progress notes, laboratory results, and discharge summaries may all have independent databases. This complexity is further multiplied by interactions with outpatient clinics and health authorities across provincial or state boundaries.

The incomplete adoption of electronic medical records (EMRs) illustrates the lag in digital infrastructure integration despite electronic record technology being available. The Canadian Federal Government’s Economic Action Plan provided funding to health care providers toward establishing EMRs in primary care in 2010, leading to an increase of EMR adoption [18]. A similar progression took place in the United States in 2014 [19]. Despite this, there continues to be reliance on paper files in both primary care clinics and hospitals [20]. If, for instance, an algorithm in an emergency department requires baseline laboratory markers for a patient from their family physician, then standardization and likely digitalization of the input data is required.

There are currently 11 certified EMR vendors and 12 EMR products in Ontario [21]. Although hospitals often have a primary vendor, they often employ a variety of disparate EMR products in affiliated practices [21]. In theory, digitization of health care data would provide an abundance of high-quality data for AI research. However, EMR vendors operate in silos and use their own approach to storing data. To implement an AI product in practice may necessitate creation of a completely novel data pipeline to aggregate records across different databases. There are attempts at standardization including the “EMR Content Standard” by the Canadian Institute for Health Informatics [22]. This introduces a content standard for EMR data entry, but levels of prioritization of the standard differ across provinces, and no standard EMR data entry has been universally adopted, resulting in the persistence of difficulty in coalescing data to be usable by AI.

For AI technology to be successful, patients must consent to its use and trust the safety of the technology. A recent public opinion survey in the United States on AI indicated that data privacy was considered to be the most important issue [23]. Privacy concerns and restricted access limits access to a diverse and large sample size, which is necessary for an AI algorithm to be validated and implemented in clinical practice [24]. A diverse data set is also crucial to guarantee adequate representation of patient cohorts in AI algorithm training [25]. There are approaches to overcome these barriers including federated learning, where a model is shared across different centers for training without exporting data [24]. However, these approaches require universal agreements regarding scope and are currently not standard of practice.

## Limitations of Technological Literacy

Medical AI applications have become increasingly relevant at an accelerated rate, though the lag in technological literacy of health care professionals for AI technology exceeds the expected social and cognitive lag of adapting new technology [26]. One challenge is that there is currently no standardized curriculum for AI education nor are there any relevant accreditation requirements within most medical doctorate programs [27]. This gap is significant as health care professionals are the main users of medical AI applications and will have to be responsible for appropriate usage of AI applications [28].

Despite a recent surge in interest in training health care trainees in AI, universal integration of AI education into current health care training is a nontrivial challenge. Medical training is dense and rigorous with significant demands on trainees and staff [29]. Implementation of such a curriculum also requires specific faculty expertise. Even with qualified educators available, there is the challenge of selecting the correct depth and breadth of topics required for medical trainees.

Without appropriate medical AI education, health care professionals may not be adequately equipped to navigate the potential ethical and legal implications of AI in health care. The flexibility that health care providers have in using their judgement to make clinical decisions tailored to an individual patient, using contextual understanding of interpatient and intrapatient variations, is essential to medicine. This process may be impeded if the end user lacks the basic digital literacy to understand the limitations of such applications of AI; for instance, deciding when to override an AI analysis in favor of contextual clinical judgement or vice versa. However, acquiring digital competency in AI applications may imply time away from service for health care providers and extra study workload for health care trainees, in addition to growing medical knowledge. Other challenges that contribute to the gap in technological literacy include lack of awareness of digital knowledge required for health care, lack of equitable access to AI education, and limited trust in AI applications in health care.

Medical applications must be well performing, trustworthy, transparent, interpretable, and explainable. Interpretation of AI models requires technical training, making it difficult to assess its performance. This is especially true in complex AI models such as deep neural networks, where it is not often possible to examine what features are used to compute the output, creating a colloquial “black box” algorithm. The gap in technological literacy among health care professionals, which is further hindered by the difficulty in implementing AI literacy training of an appropriate scope, prevents many AI applications from advancing beyond the proof-of-concept “computer-side” stage to bedside application [30].

## Limitations of Ethical Challenges

In the presence of errors by AI decisions, there lies challenges not only in identifying liability but also in quality improvement analysis. Harm caused by AI may be due to several reasons in the pipeline, such as poor data stewardship, incomplete mathematical constraints resulting in an inaccurate model, or inappropriate usage by a clinician [31]. For instance, if an AI algorithm misdiagnoses a patient, causing an adverse event, is the error associated with data collection that was not representative of patient characteristics, with inadequate algorithm development resulting in computations that produce an inaccurate prediction, or with health care administration for deciding to use an AI product? Traditional quality improvement analysis in medicine, such as cause-effect analysis, may be insufficient because it lacks a 1-dimensional cause-to-effect pathway, particularly with multiparametric AI models such as neural networks, which contain millions of computational kernels [32]. Interdisciplinary collaboration between data scientists, data stewards, clinicians, and health care workers is crucial to developing a risk liability and quality improvement system before AI can serve as a medical decision maker.

Additionally, substantial data bias may lead to unforeseen disparities in patient care as AI may stratify based on unintentional subgroups. Gichoya et al [33] observed that chest x-ray AI models can be used to predict patient’s race with image features physicians were unaware of. The implication is that bias is unavoidable even when looking at data that appears agnostic, such as chest x-rays. This may further encourage health care disparities if the model makes decisions directly correlated with race or gender. There is then a utilitarian conflict of beneficence in deciding the extent to which it is acceptable to use an AI algorithm that may be more accurate and benefit certain subgroups at the expense of others; for instance, triaging resources for subgroups that AI can accurately analyze. There is also a deontological conflict to adhere to nonmaleficence. If we know there is a high likelihood of increasing disparity despite the beneficial aspects of AI, the application of AI would be unethical.

Hence, AI poses unique ethical issues due to limitations of transparency and inherent potential for harm when used as a decision maker. AI is capable of identifying hidden features within data that can be leveraged to improve decision-making, but it is not without potential risk and needs to be deliberated by all stakeholders involved in the process.

## Conclusions

Implementation of AI in medicine faces barriers of regulatory approval, performance, compatibility of digital infrastructure, and shared multidisciplinary collaboration. Although AI shows potential in improving quality of life for patients by enhancing decision-making and tasks carried by health care professionals, the adoption of AI is likely incremental rather than a stark change in standard of care.

## Abbreviations

AI: artificial intelligence
CNN: convolutional neural network
EMR: electronic medical record
FDA: Food and Drug Administration
